# Imprinting as Basis for Complex Evolutionary Novelties in Eutherians

**DOI:** 10.3390/biology13090682

**Published:** 2024-08-31

**Authors:** Maximillian Schuff, Amanda D. Strong, Lyvia K. Welborn, Janine M. Ziermann-Canabarro

**Affiliations:** 1 Next Fertility St. Gallen, Kürsteinerstrasse 2, 9015 St. Gallen, Switzerland; 2 Department of Anatomy, Howard University College of Medicine, 520 W St. NW, Washington, DC 20059, USA

**Keywords:** evolutionary innovation, canonical genomic imprinting, non-canonical imprinting, allele-specific expression, placenta, brain

## Abstract

**Simple Summary:**

Imprinting is an epigenetic phenomenon that results in the parental-specific expression of genes. This mechanism is important for the proper development of the placenta and the brain, and influences the physiology of individuals. Any changes in gene dosage and imprinting can lead to developmental defects. While many theories address some aspects of the evolution of imprinting, none of them addresses all of them. Furthermore, imprinting is not simply the silencing of an allele, but it is a very complex mechanism that is highly regulated via several mechanisms, as, for example, methylation, acetylation, or phosphorylation. Here, we review several theories of imprinting evolution, types of imprinting regulation, and effects of imprinting.

**Abstract:**

The epigenetic phenomenon of genomic imprinting is puzzling. While epigenetic modifications in general are widely known in most species, genomic imprinting in the animal kingdom is restricted to autosomes of therian mammals, mainly eutherians, and to a lesser extent in marsupials. Imprinting causes monoallelic gene expression. It represents functional haploidy of certain alleles while bearing the evolutionary cost of diploidization, which is the need of a complex cellular architecture and the danger of producing aneuploid cells by mitotic and meiotic errors. The parent-of-origin gene expression has stressed many theories. Most prominent theories, such as the kinship (parental conflict) hypothesis for maternally versus paternally derived alleles, explain only partial aspects of imprinting. The implementation of single-cell transcriptome analyses and epigenetic research allowed detailed study of monoallelic expression in a spatial and temporal manner and demonstrated a broader but much more complex and differentiated picture of imprinting. In this review, we summarize all these aspects but argue that imprinting is a functional haploidy that not only allows a better gene dosage control of critical genes but also increased cellular diversity and plasticity. Furthermore, we propose that only the occurrence of allele-specific gene regulation mechanisms allows the appearance of evolutionary novelties such as the placenta and the evolutionary expansion of the eutherian brain.

## 1. Introduction

The process called (genomic) imprinting [1] is an epigenetic phenomenon that causes allele-specific gene expression in a parental-specific manner, namely maternal and paternal expressed genes (MEGs and PEGs), in a diploid organism. Imprinting-related gene expression may occur throughout the body but can also be restricted in a tissue-, cell-, and life stage-specific manner. Imprinting results in parental-dependent monoallelic expression of protein-coding genes, intronless retrogenes, retrotransposon-related genes, and various RNA genes, reviewed by [2]. Within the mammalian genome, imprinted genes are mostly clustered at specific conserved chromosomal regions. These clusters often possess a common control element called the imprinting control region.

In species with imprinting, the embryonic development needs genetic contributions from both sexes, commonly designated as mother (maternal specimen) and father (paternal specimen). Euploid embryos of only maternal (androgenotes) or paternal (gynogenotes) origin result in embryonic lethality and reveal characteristic and somewhat complementary developmental failures [1,3]. Epidemiologic studies in humans and animals indicate that alterations in imprinting may account for diseases and also for post-implantation pregnancy failures [4].

Until recently, it was thought that of the approximately 20,000–23,000 human protein coding genes [5], only a few hundred genes are imprinted [6]. An imprinting failure of a single gene can result in severe syndromes characterized by distinct clinical features affecting growth, development, physiology, and many other issues [7]. Most prominent examples of imprinting disorders are the Angelman and Prader–Willi syndromes (15q11-13 imprinting disorders) and Beckwith–Wiedemann and Silver–Russell syndromes (11p15.5 imprinting disorders), summarized by [8]. Additionally, a wide range of malignancies and neurological disorders are suggested to be caused by disturbed parental origin-specific gene expression.

Classical genomic imprinting, also known as canonical imprinting, is accomplished by chemical modification of CpG (Cytosine-phosphate-Guanine) islands of already primary or in the germline present differentially methylated regions (DMRs), reviewed by [9]. DNA methylation has various functions, including control of transcription, transposon repression, and X-chromosome inactivation (XCI). In canonical imprinting, the imprinting control region contains at least one DMR and controls one or few proximal genes, as imprinted genes regularly form clusters. The control is achieved via mechanisms involving long non-coding RNAs (lncRNAs), insulators, histone modifications, and higher order chromatin structures, summarized by [10]. The DMR represents different methylation statuses among different cells, tissues, or individuals, and harbors the CpG sites with parent-specific DNA methylation, reviewed by [11]. The DMRs possess cis-regulatory modules, regions of non-coding DNA, which regulate the transcription of neighboring genes. In consequence, MEGs and PEGs cannot be co-expressed in cis-configuration due to DMRs in the genome.

DNA methylation is found across the tree of life, reviewed by [12]. The mechanism in social insects, which is often also called imprinting, represents not true imprinting as it is not a silencing or inactivation of an allele or chromosome. For example, in Hymenoptera (wasps, bees, ants), males are haploid as they inherited only the maternal chromosomal set [13]. The mechanisms of imprinting facilitated by methylation of specific DNA regions are to some extent very similar between plants, reviewed by [14], and therian mammals, reviewed by [15,16], which is a great example of convergent evolution, reviewed by [17].

Since many imprinted genes in mammals are found in the placental development, it could be assumed that imprinted genes are absent in oviparous animals. For example, in most mammals the placenta-related genes *mannose 6-phosphate/insulin-like growth factor 2 receptor (M6P/IGF2R*); [18] and *insulin-like growth factor 2 (IGF2*); [19] are monoallelic expressed, i.e., imprinted [20,21]. However, in chickens they are biallelic expressed, i.e., not imprinted [22]. Therefore, the genes are present in non-mammalian animals, but the mechanism of imprinting evolved in the last common ancestor of mammals or eutherians. However, within the different mammalian lineages, different selective forces, resulted in independent evolution of imprinted domains, reviewed by [23].

Imprinting is also found during brain differentiation, see part 7 of this manuscript [24,25]. Due to its occurrence in various tissues and life stages from fertilization to adulthood, it was suggested by several scientists that imprinting was evolutionary adaptive and might have accelerated speciation [26,27]. At first glance, imprinting might not fit into the Darwinian evolution theory. In general, both haploidy and diploidy bring advantages and disadvantages for an organism. Haploidy profits from lower mutation load and faster spread of beneficial alleles, but it faces the direct impact of mutations [28]. Diploidy benefits from the minimization of mutation impacts, and allows recombination in sexual reproduction and increasing offspring diversity [29], but it requires a complex cellular architecture and bears the danger of error-prone mitosis and meiosis. Imprinting represents a de facto functional haploidy for distinct genes without losing the drawbacks of diploidy (see part 3 of this manuscript). This prompts the question of the evolutionary significance of monoallelic gene expression of an otherwise mostly diploid multicellular organism. Numerous theories were postulated to underline the significance of imprinting (see part 2 of this manuscript).

The classical understanding of (genomic or canonical) imprinting implies a DNA-methylation-dependent on-off mode of the two alleles (allele-specific silencing). However, DNA-independent, so called non-canonical, imprinting and the biased allele-specific gene expression (ASE), sometimes erroneously also referred to as non-canonical imprinting, complicate our understanding of imprinting mechanisms. The analysis of ASE is challenged by a multitude of potential biases and confounding factors (see part 6 of this manuscript). With modern transcriptome analysis technology to distinguish allelic origin of expression, the breakdown to single-cell RNA (scRNA) sequencing analysis, as well as the progress in analyzing potential epigenetic marks, it becomes obvious that biased ASE substantially exceeds the few hundred known canonical imprinted genes. The importance of these findings is uncertain as a significant increase in the number of imprinted genes would strain the current imprinting explanation approaches.

We will show here that while all theories partially explain certain aspects of imprinting, they do not fit for all its issues. Additionally, we review the current state of knowledge regarding canonical and non-canonical imprinting, biased ASE as well as XCI, and their underlying mechanisms. We aim to shortly discuss potential underlying mechanisms for imprinting evolution in eutherians, its role in diseases, and during the development and evolution of the placenta and brain. Finally, we speculate that the evolutionary rise of imprinting represents a crucial driver for evolutionary novelties.

## 2. Theories of Evolution of Genomic Imprinting

There are, besides the **prevention of haploidization**, various theories regarding the evolutionary origin and importance of genomic imprinting in a broader context. Most prominent is the **kinship (parental conflict) theory** by David Haig, reviewed by [30,31], that postulates an intragenomic conflict of sexes on the resource control of the offspring. This theory suggests that with strict monogamy, there is no conflict between matrigenes and patrigenes in the offspring, but, with increased polygamy, conflict within sexes might increase. Thus, paternal alleles are predicted to underly selection pressures that increase resource allocation from the mother towards the offspring, i.e., pregnancy, birthing, and nursing bears heavy costs to the placental mammalian mother at the expense of potentially half-siblings. Maternal alleles in this scenario would be selected for a more equal resource distribution amongst offspring as well as providing sufficient allocations for maternal survival. For example, imprinted genes impact maternal care (*Mest/Peg1*), pup behavior (*Magel2*, *XLas*), and thermogenesis as essential prerequisites for neonatal survival in mice. Several PEGs promote fetal growth in mice (e.g., *Igf2*, *Dlk1*, *Peg1*, *Kcnq1ot1*), whereas MEGs (e.g., *H19*, *Grb10*, *CDKN1C*) suppress it, e.g., [32]. However, many genes have an imprinted expression pattern, even when parental care is theoretically no longer needed or received. Additionally, the theory of maternal resource allocation within the kinship theory is challenged as most paternal disomies (both sets of chromosomes from paternal individual) are associated with either reduced growth or no phenotypes, and the fact that monotremes, which have no imprinting, also display relevant offspring care, e.g., via lactation. Yet, the observation that evolution is a continuous process and that the number of imprinted genes increases from metatherian to therian animals [33] might be an argument in favor of Haig’s theory.

Another point which cannot be explained by Haig’s theory is the predominance of MEGs in mice but PEGs in humans [34]. The former observation could be explained by the **coadaptation theory**, postulating that, in species with extended maternal care, the offspring have higher fitness if they resemble their mother more. This might be a reason why MEGs are favored as they facilitate the coadaptation of maternal and offspring tissues [35,36]. The coadaptation theory explains the imprinting process as a gain of social interactions, but it does not explain why and how PEGs evolved, nor why there is a dominance of PEGs in humans which have a longer maternal care than mice have.

The **“Enhanced Evolvability” model** suggests that imprinting enables differential selective pressures on the two alleles and thereby maintains genetic variation over evolutionary time by providing a mechanism to shield a subset of alleles from short term periods of strong directional selection [37]. That leads to the question of why imprinted genes seem only to be present in species with a high investment in their offspring (mammals and angiosperms).

The **prevention of parthenogenesis** is one of the earliest theories for imprinting [38], which is impressively documented by nuclear transplantation experiments in mice [39]. Parthenogenesis is time and energy saving and favorable when the environment is stable, but it results in lack of genetic variation. Even in imprinting-free species, such as birds and reptiles, parthenogenesis is rather the exception than the norm, reviewed by [40]. So, why has such a complex regulatory machinery evolved?

The **“Ovarian-Time-Bomb” hypothesis** includes an individual aspect with the prevention of parthenogenesis. It postulates that imprinting of growth-enhancing genes may have been underlying long-term adaptive selection pressure from females by protecting it from potentially pathological parthenogenic development of trophoblastic diseases [41,42]. These diseases are rare pregnancy-related disorders that result in the development of tumors. The “Ovarian-Time-Bomb” hypothesis considers the parallels of embryo implantation and tumor growth, such as immune evasion and cell invasion, and also that placental invasion is correlated with vulnerability to malignancy [43]. However, it is speculative whether imprinting represents a final control mechanism to prevent these pathological conditions. Again, this theory fits for some genes (i.e., *IGF2*), but why are so many genes imprinted while theoretically one or few genes would be sufficient? Second, similar to Haig’s kinship theory, why are not all growth-affecting genes imprinted? And third, why are even genes imprinted that are not involved in early embryogenesis or why are some genes, such as *UBE3A*, only imprinted in tissue-specific manner?

Barlow [44] postulated that imprinting might have evolved from a host defense mechanism that utilized DNA methylation to silence invading viruses and to defend against the mutagenic potential of transposable elements (TEs), the so-called **host defense hypothesis**. This was among others based on the observations by Rudolf Jaenisch’s group, that retroviruses introduced into the mouse genome prior to implantation almost always became heavily methylated by mid-gestation [45]. In humans, almost half of the genome is made up of TEs and about 8% consists of endogenous retroviruses, most of them are silenced, reviewed by [46]. Two main host defense mechanisms are involved in transcriptional silencing of TEs: (1) KRAB-ZFPs (Krüppel-associated box zinc finger proteins) that are involved in imprinting mechanisms in the female germline [47] and (2) PIWI-interacting RNA (piRNA; PIWI = P-element Induced WImpy testis) in the male germline [48].

Several genes such as *Peg10*, essential for placental development, and related genes such as *PEG11/RTL*, are present only in placental mammals and originated from long terminal repeat retrotransposons [49], reviewed by [50]. Additionally, there appear to be strong correlations between the evolutionary occurrence of foreign elements in the genome and genomic imprinting, summarized by [51]. Most DMRs are suggested to be derived from genome insertion events. Thereby, imprinting might be a result of DNA methylation and subsequent silencing to shield the organism from its adverse effects. However, why did imprinted regions persist when the therian/eutherian host gained evolutionary advantage of the foreign DNA? Additionally, this theory is incapable of explaining why imprinting is limited to placental mammals, reviewed by [52], and angiosperm plants.

**Figure 1:**
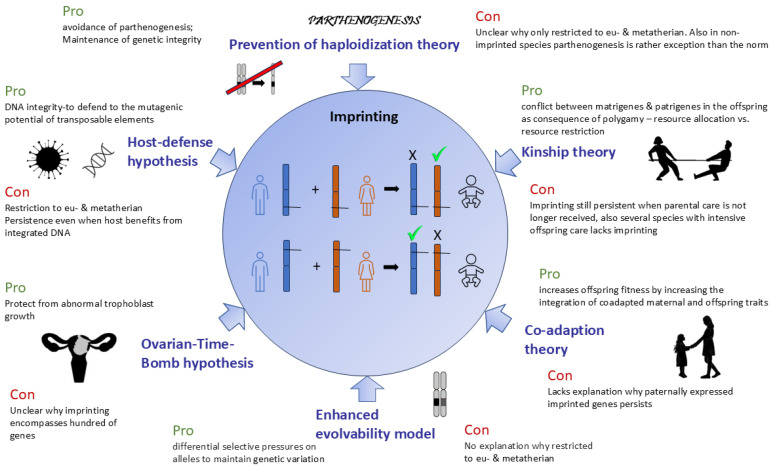
Overview of most prominent theories explaining the evolution of genomic imprinting including their strengths (pro) and weaknesses (con). The center of the figure shows the chromosome in the gametes that will be inherited by the offspring (baby silhouette on the right). The lines crossing the respective chromosomes are alleles (genes), which are imprinted in the offspring. The top represents the silencing of the paternal allele, while the bottom represents the silencing of the maternal allele. Around the circle can be found the respective theories regarding the evolution of imprinting as discussed in detail in the text (part 2 of this manuscript). For each of the theories, one representative argument for (pro) or against (con) is provided as none of the theories explain all phenomena of genomic imprinting.

In summary, all the theories presented here explain certain, but not all, aspects of imprinting (Figure 1).

## 3. Imprinting from the Vantage Point of Diploidy and Haploidy

Imprinting results in functional haploidy of distinct genes and thereby obliterates benefits of diploidy. So, why has imprinting evolved?

Both frequency of mutations and environmental dynamics have an impact on the strategy of procreation. Di- or polyploidy has several advantages including vigor effects due to the recombination of different parental genomes and gene redundancy allowing for the masking of recessive mutations. Further, in case of DNA damage, these de novo mutations can be corrected as the normal allele can be used as blueprint. The costs are higher investments for DNA packaging and higher vulnerabilities due to mitotic and meiotic errors and epigenetic instabilities.

Humans are fundamentally diploid, yet deviations from diploidy can be observed in different body cells and throughout the body [53]. This includes megakaryocytes, hepatocytes, monocytes, Purkinje cells, lactating mammary cells, and the placental bed giant cells [53]. Several advantages have been proposed for XY-ploidy including a higher metabolic activity, higher resistance against mutations and damages, and revealing a differentiated more stable state, reviewed by [54].

Haploidy in vertebrates is physiologically restricted to mature germ cells because of meiosis. Several observations in mammals support that haploidy is not favored, except in gametes. First, a significant proportion of human haploid embryonic stem cells become diploid over time by endoreplication [53]. Second, parthenogenetic mouse blastocysts formed by artificial activation of haploid oocytes often contain mixtures of haploid and diploid cells [55,56]. Murine haploid embryonic stem cells have some capacity to differentiate, but they struggle to differentiate into haploid somatic cells due to a required dosage effect (see next part of this manuscript) [57,58,59]. Third, post-blastocyst development of haploid embryos often fails. For example, bovine androgenetic embryos have limited ability to develop to the blastocyst stage due to anomalies in expression of genes from the X-chromosome and genes involved in genomic imprinting [60,61]. Therefore, imprinting, XCI, and haploidy are inevitably linked to each other, leading to abnormal developmental fates and, therefore, appear to be some natural barriers of mammalian haploidy, reviewed by [62].

Overall, genomic imprinting might be crucial to prevent parthenogenesis and development of potentially uniparental diploid or haploid organisms, but it also regulates the dosage of several genes.

## 4. The Importance of Dosage Compensation

**Figure 2:**
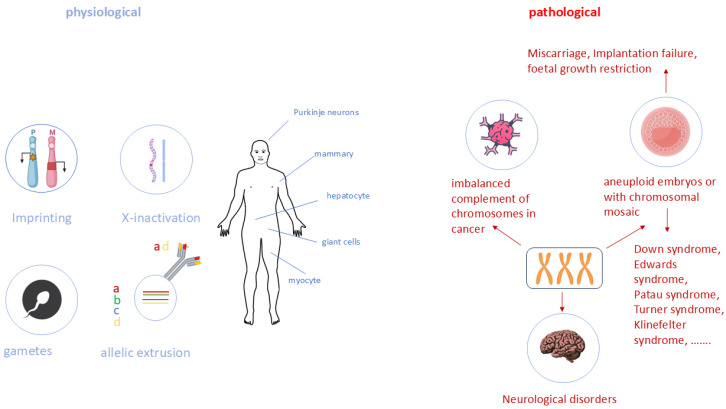
Overview of gene dosage deviation in humans in physiological (blue, **left**) and pathological (red, **right**) conditions. There are several physiological, i.e., normal, ways of regulating the dosage of a gene. Imprinting is the inactivation of one parental allele/gene, while X-inactivation deactivates one chromosome and all genes on it. Gametes (sperm, ovum) also carry different genetic material from the parents (e.g., mitochondrial DNA is exclusively maternal), while allelic extrusion changes the amount of gene expression by silencing one allele independent of the parental origin. More details are provided in the text. a, b, c, and d represent alleles on chromosomes; M—maternal chromosome; P—paternal chromosome.

Copy number variations are linked to gene dosage deviations and present in healthy individuals, but are also a common cause of diseases, development abnormalities, and miscarriage (Figure 2), reviewed by [63]. Dosage compensation per se is the process by which the expression levels of sex-linked genes are altered in one sex to offset a gene expression between females and males of a heterogametic species, reviewed by [64]. Different mechanisms have evolved to guarantee this, namely hypertranscription in flies, reviewed by [65], hermaphrodites are downregulating both X-chromosomes in nematodes, reviewed by [66], and XCI in meta- and eutheria; [67], reviewed by [68].

In humans, the X-chromosome contains the most immune-related genes of the whole genome [69]. Therefore, the escape of some X-linked genes from XCI is an assumed reason for the female bias of autoimmunity and somewhat confirmed by higher incidences of distinct immune diseases such as systemic lupus erythematosus or Sjögrens syndrome in Klinefelter (XXY) men [70]. Immune genes also play a role in placentation. Patients with XCI-escape-dependent disorders can conceive, but it might contributes to pregnancy complications [71].

Gene dosage effects of trisomy result in a 50% increase in the mRNA (messenger RNA) levels of several trisomic genes [72,73]. Due to the similarities between XCI and imprinting it could be hypothesized that in an ancestral species the mechanisms involved in XCI were adopted to autosomal regions and thereby presenting the basis for genomic imprinting, reviewed by [74]. Yet, it is unknown why some genes show dosage compensation while others do not. One reason could be that some genes only need to pass an expression threshold to guarantee proper function while others depend on expression gradients. Again, others might need a precise regulation as lower and higher gene expression could have fatal consequences, e.g., an increase in the imprinted *PEG10* gene causes cancer and a decrease growth inhibition [75].

The fine-tuning of gene expression levels via imprinting is further supported by the fact that both loss and gain (with exceptions) of the function of imprinted genes lead to syndromes. For example, the *PEG11*/*RTL1* gene functions in eutherian placentation and brain development [76], and is mainly responsible for the imprinting-related Kagami-Ogata and Temple syndromes [77,78]. Over- and underexpression results in placenta abnormalities and a variety of neuromuscular and/or psychiatric symptoms [78]. The loss or gain of methylation on the human imprinting center 1 (*IC1*) region on chromosome region 11p15 results in the overexpression or repression, respectively, of *PEG3* [79]. *PEG3* is a positive regulator of the cancer aggressiveness and angiogenesis *IGF2* gene. Misregulation of the imprinted *IGF2* results in Beckwith–Wiedemann or Silver–Russel syndromes [80,81] and overexpression of *IGF2* is a hallmark of various embryonic tumors, including Wilms tumor [82].

The zinc finger gene *Zdbf2* is paternally expressed in most embryonic and adult tissues in human and mouse [83], but in the placenta it is biallelic expressed. In mouse, *Zdbf2* controls neonatal growth via the hypothalamus, but here the precise gene dosage is important but not the parent of the origin dependent expression [83], a phenomenon called allelic extrusion. The same was demonstrated for the murine *Delta-like homologue 1* gene (*Dlk1*), which regulates proliferation and differentiation of multiple stem-cell populations [84]. Deviations from a balanced expression leads to either premature cell differentiation or increases proliferation of the precursors, and to perinatal lethality, but not flipping the parental origin of expression [85,86].

*Dlk1* is paternally expressed in the brain’s pellucid septum, but it is biallelic expressed in the ventral striatum’s neurogenic niches and in the subgranular zone of the dentate gyrus nonneurogenic regions including the ventral tegmental area [84,87]. In mouse, the proper *Dlk1* dosage modulates the stem cell commitment with the consequence of lifelong impact on pituitary gland size und its hormone production [88]. Moreover, *DLK1* is expressed in many common malignancies and is often associated with poor prognosis [86]. This all indicates that *Dlk1* needs a fine-tuned gene regulation in a cell specific manner.

The *ubiquitin protein E3A ligase* gene (*UBE3A*) is mostly biallelic expressed. However, *UBE3A* is maternally expressed in the neurons of the central nervous system, but without a dosage reduction [89]. The activity-loss of the maternally inherited *UBE3A* and paternally inherited overexpression of *UBE3A* (PatUPD15q) cause the Angelman syndrome with a typical autistic phenotype [90]. Mice overexpressing *Ube3a* display autism-related behavioral traits and impaired neuronal homeostatic synaptic plasticity driven by *Ube3a*-mediated retinoic acid repression [91,92]. Imprinting of the *Ube3a* gene in neurons is achieved through increasing expression of the maternal *Ube3a* allele in step with decreasing expression of the paternal *Ube3a* allele [89]. This relatively constant gene expression holds among tissues and between eutherian and metatherian mammals, despite different imprinting states. Therefore, imprinting of *UBE3A* in neurons is not to reduce the gene dosage but to regulate some other, still unknown, aspect of gene expression or function [89]. An indication might be provided by the observation that mice overexpressing *Ube3a isoform 2* in excitatory neurons have alterations in the forebrain, hippocampus, striatum, and amygdala, as well as elevated anxiety-like behavior responses, and learning and memory impairments [93]. In sum, constant gene expression among tissues and between eutherian and metatherian mammals is owed to tissue and species-specific critical expression thresholds.

## 5. Monoallelic Expression in Development and Disease

DNA gene coding information is transcribed to mRNA, which is later translated into an amino acid sequence and, with additional components, forms a protein. The understanding of transcriptional and, in the case of protein-coding genes, translational regulations is important because differences in dosage and spatial and temporal expression may be the major cause of phenotypic differences. Generally it is assumed that, with a few exceptions, biallelic gene expression at similar levels is the norm in diploid eukaryotic organisms [94].

The most prominent type of imprinting is the X-chromosome inactivation (XCI) in metatherian and eutherian females. Although there are differences between both events of transcriptional silencing, similarities between XCI and autosomal genomic imprinting have been noted for a while [95,96]. Both processes are highly organized with epigenetic modifications and, in placental mammals, the involvement of lncRNAs. Failures in both often result in pathological conditions. In marsupials, XCI is paternal determined throughout the body, therefore representing a parental monoallelic expression [97]. In the extraembryonic tissues of some eutherian species, XCI of the paternal X-chromosome remains in contrast to randomized XCI in post-implantation development in humans [98]. A tendency towards gene clustering is observed in both XCI and in genomic imprinting, and both are absent in Prototheria. Other examples of monoallelic expressions are allelic exclusions, e.g., in B- and T-cell receptors, and neurons expressing olfactory receptors, allowing cell diversity and specificity [99,100].

With the advantages regarding allele-specific expression (ASE) analysis at a single-cell (sc) level, RNA-sequencing (RNAseq; scRNAseq) has fundamentally changed our understanding of ASE. During canonical imprinting, a complete silencing of one parent’s allele occurs in (A) the whole organism, (B) a distinct tissue, or (C) on cellular level. However, a still unknown number of genes are subjected to non-canonical imprinting (see part 6 of this manuscript), where (1) an allelic bias in each cell or (2) an allele silencing in a subpopulation of cells in the tissue might be present.

Imprinting mediated monoallelic expression is involved in many cellular processes and is essential during embryogenesis and fetoplacental development. These genes include growth factors, cyclin dependent kinase inhibitors, transcription factors, and others. For most of them, the loss of expression, but also a biallelic expression, has severe developmental consequences, summarized by [101].

Disturbed monoallelic expression is often associated with an escape of XCI that might substantially contribute to autoimmune diseases or loss of (prominent) imprinting (LOI)-related diseases such as Prader–Willi or Angelman syndromes, reviewed by [102]. Dysregulation of the imprinting pattern or LOI have also been described for several tumors such as breast cancer, glioblastoma, Wilms tumor, or ovarian carcinoma [82,103,104]. For example, in HER2-enchriched and basal-like tumors, downregulation is associated with the massive induction of biallelic expression of, e.g., *ZDBF2*, *PEG10*, *MEST*, *H19*, *IGF2*, *MEG3*, *ZNF331*, and *HM13* [105]. LOI of the *IGF2* gene is likely the main reason for increased tumor predisposition in Beckwith–Wiedemann syndrome [106]. Furthermore, the significant hypomethylation of H19-DMR region II with different expression of *IGF2* was observed in eutopic and ectopic endometrial tissues of endometriosis patients, compared to the control tissues [107]. These findings indicate that LOI occurs in malign and benign conditions. However, it is not clear whether LOI is the cause or side effect of these pathological conditions.

Several classical (canonical) imprinting-related disorders such as Angelman and Prader–Willi syndrome affect the nervous system and are acknowledged as neurological disorders. Additionally, it has been suggested that LOI might be involved in a broad range of neuropsychiatric and neurological disorders. Studies of rare copy number variants have highlighted associations between imprinted genes and the incidence of severe neuropsychiatric disorders, e.g., [108]. Others demonstrated a disproportionately high occurrence of imprinted gene expression in the brain and hypothalamus (see part 7 of this manuscript). Several genes such as *Ndn*, *Grb10,* and *Ube3a* are imprinted strictly in neuronal cells [89], reviewed by [109]. For several other genes, such as the imprinted *GABRB2* gene, there seems to be a connection to bipolar disorder, epilepsy, autism spectrum disorders, Alzheimer’s disease, and dementia, e.g., [110]. Therefore, LOI might be linked to neuropsychiatric and neurological disorders, but more research is needed.

## 6. The Broader Picture of Allele-Specific Expression

Genomic imprinting is one of several epigenetic-based regulation mechanisms that results in different expressions of maternal and paternal alleles. The phenomenon of allelic exclusion is not restricted to imprinting and XCI. It is well known in lymphocytes (B- and T-cells), where it allows the expression of a single type of immunoglobulin [111], and olfactory receptors, where protocadherins are expressed [112]. The monoallelic expression is suggested to determine the neuron’s sensitivity, allowing mono-specificity of immune cells, and shaping individuality [113].

**Figure 3:**
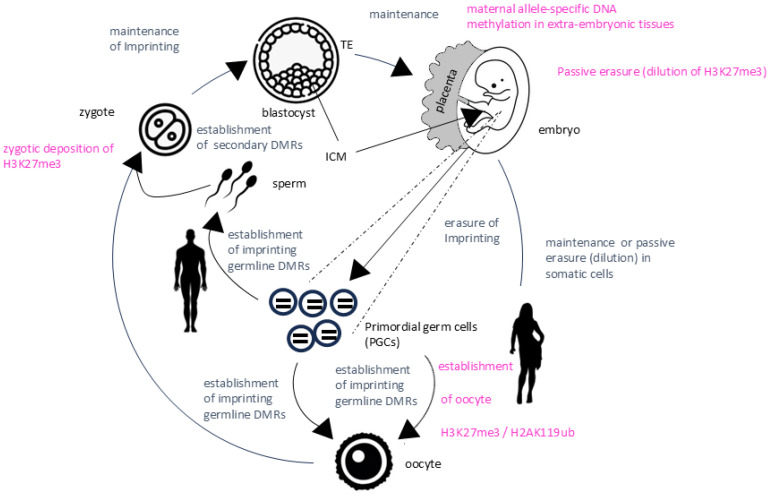
Life cycle of imprinting during development. Blue: canonical imprinting, pink: non-canonical imprinting; DMR: Differentially methylated region; ICM: inner cell mass; TE: trophectoderm.

The classical understanding of imprinting involves differentially methylated regions (DMRs). A family of DNA methyltransferases (DNMTs) catalyzes DNA methylation (Table 1). With new methodologies, more DMRs are reported, implying that imprinting is wider spread in the eutherian genome than previously believed [114]. Additionally, besides the canonical imprinting based on parental DNA methylation in the DMR, a DNA methylation-independent, so-called non-canonical imprinting exists [115]. There is some inconsistency in using the term “non-canonical imprinting”. It is often used redundantly regarding allelic bias in expression while canonical imprinting involves complete silencing of one allele. We would, therefore, clearly separate these two definitions. That is, non-canonical imprinting refers to the difference in the silencing mechanism mediated by maternally histone modifications (e.g., by histone 3 lysine 27 trimethylation (H3K27me3)) (Figure 3) [49,116,117]. Additionally, non-canonical imprinting is restricted to embryonic stages, in particular extra-embryonic tissues, while allele-specific expression (ASE) bias is also reported in tissues of adults.

ASE or parental expression bias is the significant bias of genes to express one parental allele at a higher level than the other one. ASE is also observed in adult somatic tissues and was demonstrated for several genes (e.g., Ddc, TyrH) in distinct subpopulations of brain neurons in mice [125,126]. RNAseq-based analyses resulted in 186 autosomal genes with a significant expression bias of either the maternal or paternal allele; of those, 142 have not been previously annotated ([125]. ASE is associated with histone modifications H3K9me3 (trimethylation) and H3K9ac (acetylated) [125] but the detailed mechanisms of ASE regulation are currently almost unknown (see Table 2).

There are likely more imprinted genes than currently known and imprinting becomes even more complex considering that some genes are regulated by both canonical and non-canonical imprinting, reviewed by [51]. In the last decade, several genes were found to be imprinted although they lack detectable DNA methylation differences between parental gametes. These genes revealed an imprinting pattern that is dependent on histone modifications by dimethylation (H3K9me2) and/or trimethylation (H3K27me3) [128]. By 2019, over 200 genes were reported in mice, summarized by [129], and in 2021, an allele-specific transcriptomics and uniparental methylome profiling analysis found 111 genes with previously undescribed parent-of-origin-specific bias in murine blastocysts [117]. Of 71 genes with parent-of-origin-specific expression with an allelic ratio of at least 70:30, only 16% were found to be associated with DMRs, while most were associated with parentally biased H3K27me3 [117], i.e., parent-of-origin-specific non-canonical imprinting.

Non-canonical imprinting is tissue- and cell-specific and is essential for regulation of placenta-specific genes, summarized by [127]. Several of these genes, such as *Gab1* and *Sfmbt2* in mice, are regulated by this alternative allelic expression, reviewed by [130]. Moreover, H3K27me3 serves as a maternal imprint for the lncRNA *Xist*, triggering paternal XCI in female mouse pre-implantation embryos and extraembryonic tissues (Figure 3), reviewed by [131]. Non-canonical imprinting is poorly conserved among species, reviewed by [132], and the full extent of this histone-modification based imprinting is rather unclear, reviewed by [133]. Therefore, intensive research is ongoing and detailed functions of non-canonical imprinting remain elusive.

To sum this up, non-canonical imprinting is sometimes equated and incorrectly referred to as the phenomenon of ASE or parental expression bias. Biallelic equal expression of maternal and paternal autosomal genes is probably the most common form of gene expression, but with ASE bias a significantly higher level of expression arises from one allele versus the other [134]. The study of ASE is difficult and detailed molecular mechanisms behind it are still unclear. ASE does not necessarily correspond to non-canonical imprinting.

Histone modification is a type of epigenetic mechanism that modifies the amino (N)-terminal end of histone tails in nucleosomes. These changes include phosphorylation, acetylation, methylation, and several others [135], reviewed by [136]. The acetylation of H3, including the above mentioned H3K9ac (acetylation of lysine 9 in histone 3) is associated with the active state of euchromatin, while methylation of lysine 27 (H3K27me3) is associated with the silencing of heterochromatin. Methylation is not as straight forward as acetylation and its impact on gene expression depends not only on the degree of methylation (mono-, di-, trimethylation) but also on the location of the methylated residue, resulting in activation of the deactivation of transcription [137]. Therefore, as mentioned above, histone modification is one mechanism of controlling imprinted genes for more details, see [138].

## 7. Evolution of Imprinting in Organs

Canonical imprinting plays pivotal roles in the development and homeostasis of the placenta and brain. In androgenotes and parthenotes, both organs are the most affected ones, revealing drastic changes in size. Several genes that are biallelically expressed elsewhere demonstrate monoallelic expression in these organs or at least in distinct cell types of these organs. For example, the *UBE3A* gene is mostly biallelic expressed, but it is maternal expressed in the brain, and deficiency leads to a complete lack of expression in glutamatergic and GABAergic neurons, also known as Angelman syndrome (see part 4 of this manuscript). Other examples include the genes *Ano1* and *Gab1* that have placenta-specific imprinted expression [128]. Yet, imprinting in the placenta and the brain is likely overrepresented due to the overestimation of the number of genes with parent-of-origin-specific expression [32].

Imprinting may be evolutionarily adaptive as it is involved in placental and brain development [24,25,139], and might have accelerated speciation [26,27,140,141]. Here, we will review the role of imprinting during evolution and development of both organs, starting with the placenta as it is also involved in brain development.

(1) Placenta

The placenta is an organ that is formed by fetal membranes that are in close contact or fused with maternal uterine mucosa [142]. Using this definition in its strict sense, placentas can be found in all vertebrates, except birds, reviewed by [143]. However, while placentation evolved multiple times independently and convergently in vertebrates, it only evolved once in the last common ancestor of mammals and is in mammals linked to the evolution of imprinting. The main difference in mammalian placenta development from the placenta development in other vertebrates is the earliest differentiation of a cell lineage designated to become placental tissue, i.e., the distinction of the so-called trophoblast from the embryoblast. The trophoblast is contributing to the placenta and the embryoblast is giving rise to the embryo; for more details see review by [144].

The placenta is a feto-maternal organ [145] and the mother’s wellbeing affects the fetal environment in utero and potentially changes gene expressions, that directly influence the offspring’s development [145]. Placental dysfunction is associated with pregnancy and birth complications including intrauterine growth restriction, low birthweight, or fetal demise, pre-eclampsia, and postnatal problems, including long-term health conditions such as metabolic and mental disorders, e.g., schizophrenia [146].

During the mammalian evolution the acquisition of a placenta is closely correlated with the genomic imprinting of placenta-specific genes, reviewed by [2]. Placental structures among mammalian species are diverse [147] and imprinting is not always conserved between different species [148]. For example, there is a predominance of MEGs over PEGs in the placenta in mice [149] but a predominance of PEGs in equids [150]. Furthermore, to date, 150 imprinted genes were identified in mice but only 100 in humans, and genes that are exclusively imprinted in the placenta are often not conserved between mice and humans [148,151]. Indeed, most placenta-specific imprinted genes identified in humans are not differentially methylated in the mouse placenta [152,153]. A short gestational period in mice might increase the evolutionary selection pressure towards genes that make the placenta more efficient for the requirements of the developing offspring, which would lead to more imprinted genes in mice as compared to humans [148]. A longer gestation period reduces this pressure as it is sufficient to provide the offspring with the nutrition needed to differentiate complex tissues (e.g., brain). Another factor might be the number of offspring in a single pregnancy with typically one to two in human and up to eight in mouse, which causes increased intra-litter competition. A reduction in competition would relieve the pressure to keep placental-specific imprinting and result in less imprinted genes in human [148]. There are even variations of imprinted genes within the same species as seen in the human placenta, where there is a high retention of oocyte-derived methylation, and this placenta-specific imprinting is very polymorphic among individuals [154,155]. Taking variations in imprinting mechanism and imprinted genes in the placenta, and likely also in other organs like the brain, into account, it is evident that imprinting might have evolved in the last common ancestor in mammals, but then a variation of selection pressures resulted in the observable differences in imprinted genes, imprinted gene numbers, times in which imprinting is active, etc., reviewed by [23].

Some of the eutherian (e.g., mouse, human) imprinted genes are also imprinted in metatherians (e.g., wallaby, opossum) [156,157,158] and, therefore, are likely already imprinted in therians, the common ancestor of both. In prototherians (platypus, echidna), birds, and reptiles, these genes are either biallelically expressed or absent [20,22,33]. This implies that genomic imprinting evolved after the divergence of monotremes from marsupials and eutherians, which coincides with increase in gestational length and a more invasive placentation, reviewed by [159]. If placentation is a driver in the evolution of imprinted genes, than this would explain why many imprinted genes are predominantly or exclusively expressed in the placenta, where they regulate the nutritional supply, and placental and fetal growth, reviewed by [160,161]. There might also be a correlation between imprinting differences and placental diversity, although this is unproven. Alternatively, the evolution of imprinting could be the prerequisite for the evolution of placentation.

Because of the parallels between imprinted XCI and autosomal imprinting of placenta-specific genes [67,162], it is hypothesized that these mechanisms have coevolved with placenta appearance and depend on the recruitment of histone-modifying enzymes as well as the presence of lncRNAs [162]. This implies that the imprinting mechanisms were originally conserved in placental mammals. Later, species-specific differences in the placenta-specific imprinted regions evolved [148]. X-linked gene inactivation relies on the paternal lncRNA *Xist* to be expressed [162,163,164]. However, the monoallelic expression appears to be randomized, and in human placenta and pre-implanting embryos *Xist* expression is biallelic, while it is monoallelic in mice [96,98]. The randomized extraembryonic XCI in humans is related to the lack of autosomal placental imprinting, which led to the loss of a common mechanism in mammals. A similar lack of autosomal placental imprinting is related to lncRNAs *Kcnq1ot1* and *Air* [148,165]. In the murine orthologous region of the KCNQ1 domain, 8 out of 14 imprinted transcripts are maternally expressed in the placenta. In humans, there are only six imprinted genes expressed, indicating an evolutionary loss of placenta-specific imprinted genes.

Most placenta-specific imprinted miRNA genes are created from three evolutionary distinct chromosomal domains: C2MC, C14MC, and C19MC, reviewed by [2]. The primate-specific, paternally expressed C19MC miRNAs (human chromosome 19q13.4) are one of the most abundant expressed miRNAs in the human trophoblastic cells [166]. The rodent-specific, paternally expressed C2MC (intron 10 of *Sfmbt2* gene) promotes placental growth. The eutherian-specific, maternally expressed miRNAs within the imprinted Dlk1-Dio3 domains (human chromosome 14q32 = C14MC) limit placental growth, reviewed by [2].

Several imprinting disorders and disturbed imprinting of PEGs lead to severe abnormalities in placental development, including growth restriction, overgrowth, or other malformations. For example, Beckwith–Wiedemann syndrome is associated with increases in placental weight, while Kagami–Ogata syndrome demonstrates placental hypertrophy and placental hypoplasia [167,168], reviewed by [169]. Canonical imprinted placental genes were found to be involved in the spongiotrophoblast development and maintenance of the fetal capillaries [170]. Alterations of some human imprinted genes were linked to several pregnancy-related diseases [171,172], summarized by [173]. Several placental genes are non-canonically imprinted such as *Gab1*, *Sfmbt2*, and *Slc38a4,* and dysregulations of these genes lead to severe developmental defects in the placenta [174,175]. Also, a link between dysregulation of imprinted genes, disturbed gene expression, and hypertensive disorders in pregnancy or intrauterine growth restriction has been indicated [176,177].

The expression of imprinted genes in the placenta also influences the brain of the mother, which we address shortly in the next part.

(2) Brain

Almost half of the known imprinted genes also show imprinted expression in the brain, reviewed by [129]. These genes are expressed in the developing and adult brain and are related to several aspects of brain function, including behavior [24], reviewed by [178]. There is a regulation of imprinted genes with an enrichment of MEGs during the embryonic development, whereas PEGs are more commonly found in the adult brain, e.g., [179], reviewed by [101]. PEGs and MEGs are also differentially expressed in various brain locations. Gynogenetic cells are found throughout the hippocampus, cortex, and stratum, and androgenic cells are enriched in the hypothalamus, preoptic area, and septum pellucidum [24].

Many brain-specific imprinted genes are not imprinted in other tissues and various brain areas express different sets of imprinted genes, which implies that the parental regulation of gene dosage is important for the normal brain development and function [179,180]. Functions of imprinted genes can also be studied in gynogenic and androgenic hybrids and cell lines. Keverne and Fundele [181] demonstrated that gynogenic-wildtype chimeras develop an enlarged brain, while androgenic-wildtype chimeras develop unusual small brains.

Several neurodevelopmental processes such as cell proliferation, differentiation, neuronal migration, axonal and dendritic outgrowth, and self-renewal of neural stem cells are influenced by imprinted gene expression. In the adult brain, synaptic plasticity is affected by imprinted genes that control action potentials, synaptic transmission, and pre- and post-synaptic regulation, which results in the impact of imprinted genes on brain functions (social behavior including mother–offspring interactions; learning, memory, energy homeostasis) [182,183].

The placenta influences brain development (placenta–brain axis) and synchronizes the expression of several imprinted genes that determine the brain growth, reviewed by [178]. The placenta produces various neuropeptide hormones that are analogous to those produced by the pituitary gland and hypothalamus, including Corticotropin-Releasing Hormone (CRH), Thyrotropin-Releasing Hormone (TRH), Gonadotropin-Releasing Hormone (GnRH), and oxytocin [184,185]. There is an integrated regulation of neuropeptide homeostasis within the placenta and the brain, therefore linking the developed (maternal) and developing (embryonic and fetal) brains. Therefore, any changes in the placenta development (e.g., mutations in *Gab1*) or physiology, summarized by [129], might impact the brain development, reviewed by [186]. Furthermore, poor placentation is linked to several chronic neurodevelopmental disorders in children, recently summarized by [187].

An imbalance of PEGs and/or MEGs during brain development was suggested to be responsible for some types of autisms [90,92]. In the adult mouse brain, sex-specific parent-of-origin allelic effects were described [179]. For example, in the glutameric neurons of the female murine cortex, the maternal X-chromosome is preferably expressed. Almost 350 autosomal genes with sex-specific imprinting patterns were found in the cortex and hypothalamus [179]. In the latter, imprinted genes were mostly found in females, implicating that there might be an influence on the hypothalamic function of daughters. A specific example is Interleukin 18, which is linked to diseases that result in complex, regional, and sex-specific effects in the brain [179].

A hallmark of mammals, besides placentation, is significant and progressive evolutionary enlargement of the brain, especially in primates. Two regions were expanded largely during human evolution, the neocortex and the ventral striatum. The dorsal cortex of reptiles and the hyperpallium (wulst) in birds is a simple structure and composed of a single layer of neurons, that likely resembles an early precursor of the neocortex, reviewed by [188]. However, the largest part of the hyperpallium is homologous to the mammalian primary visual cortex [189]. During mammalian evolution, the neocortex expanded, which might be a consequence of genomic imprinting in the neocortex, e.g., [190].

A six-layered neocortex was already present in mammals, i.e., the last common ancestor of monotremes and therian animals [191,192], but premotor areas likely evolved only in eutherians, reviewed by [193]. Neocortices of eutherians have more neurons than similar-sized marsupials [192] and they evolved rapidly in primates, including humans, reviewed by [194]. In fact, humans have the largest brains of extant primates; that is, 80% neocortex with about 200 or more areas [195,196]. Of special interest is the corpus callosum, which is an evolutionary innovation of eutherians, that connects the two hemispheres of the neocortical areas [197]. It represents the major interhemispheric fiber bundle, while in monotremes and metatherian the connections between the two neocortices cross via the anterior commissure [197]. Agenesis of the corpus callosum may be correlated to incorrect data processing in the brain reflected by cognitive developmental delays/impairments, and difficulties in understanding language and social signals [198,199].

**Figure 4:**
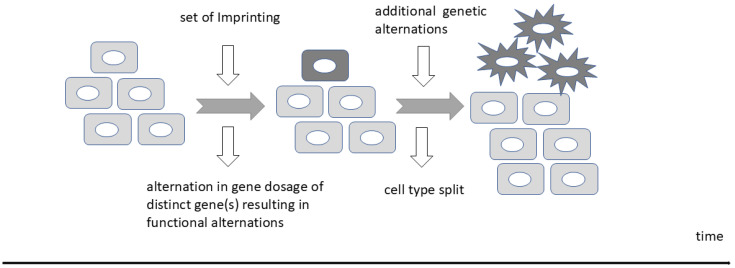
Hypothesis for imprinting as a trigger of evolutionary novelties.

Several lines of evidence support that the rapid eutherian evolution of the neocortex and corpus callosum combined with the imprinting in the brain is linked to imprinting (Figure 4). First, some neurological/neurocognitive conditions are related to loss of imprinting [76], reviewed by [200]. Second, many imprinted genes are differentially expressed during cortical neurogenesis, reviewed by [194,201], or are involved in neocortical development in animal models [190,202,203,204]. The agenesis of the corpus callosum is one of the main congenital defects in Prader–Willi syndrome, but it is also affected in Silver–Russell Syndrome [205]. Further, the *PEG11/RTL1* gene is expressed in the placenta and in eutherian-specific brain regions including the corpus callosum [76,77].

The involvement of imprinted genes in parent and offspring interaction might be evolutionary relevant. For instance, maternal *Phlda2* is expressed in the placental endocrine cells, that causes changes in hormonal levels such as placental lactogens [206]. An increase in PHLDA2 in the placenta results in a low birth weight [207]. Altering *Phlda2* expression levels in the fetal placenta changes the gene expression in the maternal brain and influences the quality of maternal care postnatally [208]. Imprinted genes also act on the brain of lactating individuals to regulate lactation or on the infant’s brain to regulate nipple attachment, suckling and feeding behavior, and likely also the emotional interactions with the mother [209,210]. A summary of imprinted genes that modulate behavior after weaning instead of growth of tissue composition is provided by [129].

Some imprinted genes regulate sleep homeostasis and circadian clock, reviewed by [129], and others alter social behavior. For example, *Grb10*-KO mice and *Cdkn1c* LOI mice with an increased expression of *Cdkn1c* are more likely to win an encounter in a tube test task of social dominance [211,212], which could indicate a functional convergence. During infant development, imprinted genes also regulate the allocation of resources to growth, fat reserves, and homeostatic mechanisms, such as temperature and glucose regulation.

In summary, imprinted genes are present during placental and brain development, in the adult brain, in the interaction between placenta and brain, and in the establishment physiological processes and behavioral interactions.

## 8. Conclusions

(1) The comparison of theories regarding the origin of imprinting demonstrates that all current hypotheses explain certain, but not all, aspects of why such a complex and vulnerable regulatory process evolved with the origin of mammals.
(2) Imprinting might represent a functional haploidy of some genes. Regarding an allele-specific expression, imprinting is not a unique feature in mammals. This aspect is also known in immune and neuronal adhesion molecules. Therefore, comparing effects of imprinting in several regions of the body could provide insights into the evolution of this mechanism.
(3) Imprinting is important in gene dosage regulation, while the origin of expression (maternal or paternal) might be irrelevant in these instances. A monoallelic expression possibly allows a better fine-tuning of gene regulation. Over- and under-expression of distinct genes can cause a phenotypic effect, which are often contrary in their phenotypic aspects. Several imprinted genes are dysregulated in cancer and other severe pathological processes. Notably, XCI and imprinting share many common features, including gene dosage compensation. Research into the similarities and differences of XCI and imprinting (silencing) of single genes potentially enables not only the understanding of dosage effects, but also of threshold effects and roles in gene regulatory networks that depend on precise dosages.
(4) Disturbed monoallelic expression is not only known for imprinting disorders. For example, an escape of XCI is regularly suggested as a reason for higher prevalence of auto-immune diseases women. LOI (loss of prominent imprinting) is associated with many congenital disorders and might be also involved in tumorigenesis, neurological disorders, and complications of pregnancy such as pre-eclampsia. For that reason, and following up on point 2 and 3 above, is it important to further investigate the mechanisms of gene regulations (dosage, imprinting), as this might lead to new treatments using, for example, CRISPR to regulate gene dosages, and therefore reduce the impact of XCI escape or LOI.
(5) Beside the classical canonical imprinting based on parent-specific DNA methylation, an additional mechanism of imprinting exists. The non-canonical imprinting is based primarily on histone modifications and was described in embryonic stages and extraembryonic tissues. However, this layer of epigenetic regulation is poorly conserved among species and the detailed role(s) of non-canonical imprinting remains to be elucidated. The description of developmental stage specific imprinted genes in mice suggests that imprinting is more complex than previously thought.
(6) The biased allele-specific expression (ASE), erroneously also designated as non-canonical imprinting, stresses the concept of a uniform biallelic expression and the current hypothesis of the occurrence of imprinting. With the implementation of single-cell expression analysis and the investigation of epigenetic modifications, future research will not only focus on the spatiotemporal expression patterns but also ASE.
(7) The placenta as an evolutionary novelty and the massive brain enlargement are the hallmarks of mammals. Both organs represent hotspots of imprinting, and several imprinting disorders are associated with neurological and placental anomalies. The placenta–brain axis signifies how the placenta modulates fetal brain development. Genomic imprinting allows a high plasticity on cells and organs, and both organs are characterized by high plasticity and their capability of fast adaptations to environmental changes. Genomic imprinting might not only contribute substantially to the plasticity of the placenta–brain axis but might be the key evolutionary driver of these organs.

## Figures and Tables

**Table 1:** DNA methyltransferases (DNMTs) functions.

DNMTs	Function	References
DNMT1	mainly maintains methylation and therefore imprints	[32,118]
DNMT2	transfer RNA methylation	
DNMT3	de novo methylation of unmethylated DNA	
DNMT3A	imprinting and methylation at major satellite repeats; maternal-specific germline DMR methylation occurs postnatally in growing oocytes but prenatally in prospermatogonia before onset of meiosis in male germline	[119,120]
DNMT3B	minor satellite repeat methylation	[121]
Dnmt3c (rodents)	methylates young retrotransposons in male germ line	[122]
*DNMT3L*	contains no catalytic domain for methylation, instead, it co-orchestrates de novo methylation; establishes genomic imprints in oocyte	[123,124]

**Table 2:** Comparison of imprinting and allele-specific expression ASE; modified from [127].

	Canonical Imprinting	Non-Canonical Imprinting	ASE
Establishment of imprinting	DNA methylation in oocyte or sperm at imprinting control region	H3K27me3 H2AK119ub	Allele-specific histone modifications (H3K9ac; H3K9me3)
Established by	Oocyte: Dnmt3a, Dnmt3L, Kdm1b, Zfp57; Sperm: Dnmt3a, Dnmt3b, Dnmt3L, piRNA, CTCFL/Prm7	PRC2, Pcg1/6-PRC1	Unclear
Maintenance of imprinting	ZFP57 and/or ZNF445 required to maintain DNA-methylation of imprinting control regions	H3K27me3 replaced by monoallelic DNA methylation	Unclear
Regulated by	Dnmt1, Dppa3, Zfp57	PRC2, Smchd1	Unclear
Set of imprinting	During oogenesis and spermatogenesis	During oogenesis	Unclear
Location	Widely distributed over the body, predominantly in neural and placental tissues	Extraembryonic and embryonic cell lineages	Widely distributed over the body, highly tissue-specific
Conservation between species	High	Low	Unclear
Clinical picture of imprinting abnormalities	Infertility, moles, miscarriage, classical clinical pictures of imprinting disorders such as Angelman, Beckwith–Wiedemann or Prader–Willi syndrome	Placental defects in murine loss of function/loss of prominent imprinting mutants with (sub)-lethality	Unclear

## Data Availability

All information and data are presented in the manuscript.
